# Cyclic stretching of soft substrates induces spreading and growth

**DOI:** 10.1038/ncomms7333

**Published:** 2015-02-23

**Authors:** Yidan Cui, Feroz M. Hameed, Bo Yang, Kyunghee Lee, Catherine Qiurong Pan, Sungsu Park, Michael Sheetz

**Affiliations:** 1 Mechanobiology Institute, National University of Singapore, Singapore 117411, Singapore; 2 School of Mechanical Engineering and Department of Global Biomedical Engineering, Sungkyunkwan University, Suwon 440-746, Korea; 3 Department of Biological Sciences, Columbia University, New York, New York 10027, USA

## Abstract

In the body, soft tissues often undergo cycles of stretching and relaxation that may affect cell behaviour without changing matrix rigidity. To determine whether transient forces can substitute for a rigid matrix, we stretched soft pillar arrays. Surprisingly, 1–5% cyclic stretching over a frequency range of 0.01–10 Hz caused spreading and stress fibre formation (optimum 0.1 Hz) that persisted after 4 h of stretching. Similarly, stretching increased cell growth rates on soft pillars comparative to rigid substrates. Of possible factors linked to fibroblast growth, MRTF-A (myocardin-related transcription factor-A) moved to the nucleus in 2 h of cyclic stretching and reversed on cessation; but YAP (Yes-associated protein) moved much later. Knockdown of either MRTF-A or YAP blocked stretch-dependent growth. Thus, we suggest that the repeated pulling from a soft matrix can substitute for a stiff matrix in stimulating spreading, stress fibre formation and growth.

## Abstract (web summary)

Cells grown on a stiff substrate are stimulated through physical cues to spread, create actin stress fibres and proliferate. Here Cui *et al*. show that cyclic stretching cells on a soft pillar substrate has the same effect as growth on a stiff substrate, and results in nuclear translocation of YAP and MRTF-A.

## Graphical abstract

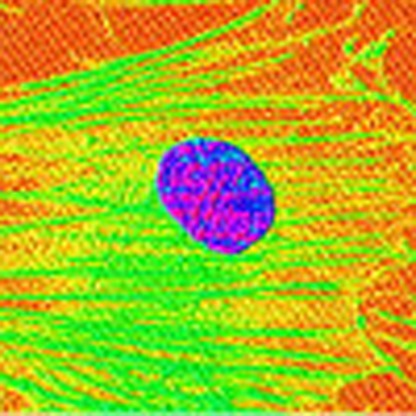

Tissue cells sense and respond to mechanical signals, such as rigidity of the extracellular matrix and mechanical stimuli, through mechanotransduction^1^,^2^,^3^,^4^,^5^. For example, cells probe the rigidity of extracellular matrix by pulling on matrix, and respond to the rigidity through adjustment of their adhesion and intracellular cytoskeleton organization^6^. This rigidity-sensing process regulates various cellular functions such as cell signalling, morphology, migration, proliferation and differentiation^1^,^6^,^7^,^8^,^9^,^10^,^11^. The behaviour of cells on soft and rigid substrates can be accurately investigated by varying the height of elastomeric pillars^8^,^9^,^12^,^13^. This approach enables the continuous measurement of the cell traction forces on a substrate^6^,^12^ and those forces are regulated in response to substrate rigidity^9^,^13^. Previously, it was reported for 500-nm pillars that the initial rigidity sensing for adhesion formation involved local myosin contraction to cause 60-nm displacements of neighboring pillars (diameter, 0.5 μm) regardless of the stiffness^14^. In the case of micron scale pillars, contractions occurred around individual pillars (dia., 2 μm)^14^. Therefore, in terms of evoking a normal cell spreading response, submicron pillars can mimic a continuous soft substrate and can capture many of the details that are not revealed in micron-scale pillars^14^.

Soft tissues often experience various mechanical stimuli, such as shear stress and mechanical strains. For example, lung tissue is cyclically stretched due to breathing, and muscles are also continuously exposed to mechanical loads^2^. Stretch sensing is generally known as an integrin-mediated pathway that couples to cell contractile activity, and thus shares many mechanotransduction pathways with the rigidity sensing process in translating the mechanical stimuli to intracellular biochemical signals^15^,^16^,^17^. The relationship between these two processes and the relative effects on cell behaviour in tissues is of general interest^6^. The effects of cyclic stretch on cytoskeleton reorganization, cell proliferation and even stem cells differentiation were reported many times^12^,^18^,^19^,^20^. Recently, it was reported that the F-actin cytoskeleton could regulate endothelial cells via dynamic focal adhesion assembly under uniaxial stretch^21^. Applying external forces to cells plated on a rigid substrate often leads to cytoskeleton reorganization and strengthening of the focal adhesions due to the cellular tendency to resist the deformation caused by force and to maintain optimum mechanical conditions^6^,^18^,^19^. At the protein level, unfolding of the adhesion protein, talin, by physical stretching can activate vinculin binding^22^, and force-dependent strengthening of focal complexes involves vinculin recruitment^23^. Experimentally, stretching has been applied to cells in different manners with regards to strain type, magnitude or duration, and many different cellular responses are induced. For example, the static stretching of vascular smooth muscle cells cultured on a soft substrate (~5 kPa) induced biphasic changes in cytoskeleton contractility, with enhanced contraction followed by the slow release of the stretching force (adaptation)^16^. However, soft tissues in the body often experience cyclic stretching rather than static strain.

To separate force effects from substrate rigidity effects, we used 2-μm long pillars (500 nm in diameter) that will have a roughly constant rigidity for about 300 nm of displacement^24^. In this study, we observed that when fibroblasts that required a rigid substrate for growth were plated on such soft nanopillar arrays, they did not spread and did not grow even after a static stretch. However, with cyclic stretching of the substrate, the cells spread and grew as if on a rigid substrate. In addition, translocation of both MRTF-A (Myocardin-related transcription factor-A) and YAP (Yes-associated protein) to the nucleus might be involved in this process.

## Results and Discussion

### Cyclic stretch increases cell spreading on soft nanopillars

**Figure 1:**
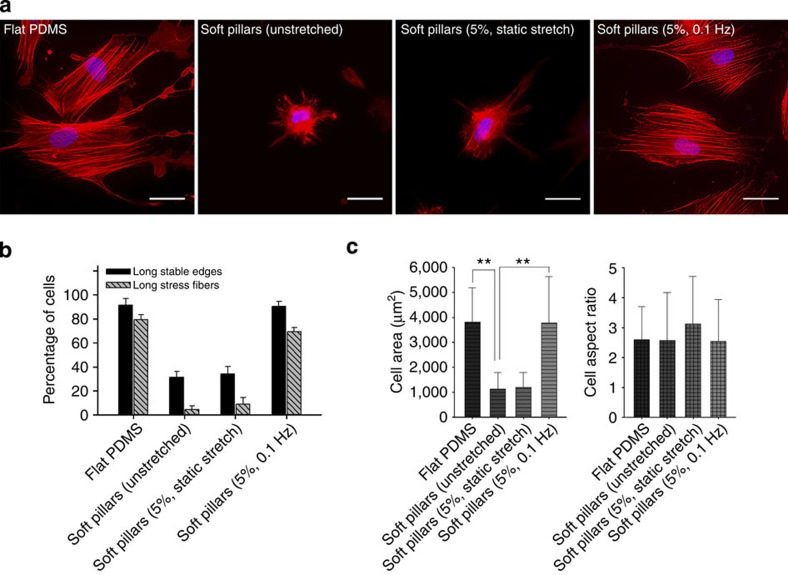
Effect of substrate rigidity and stretching type on cell spreading and stress fibre formation. (**a**) Representative images of PMEFs on flat PDMS (polydimethylsiloxane) and soft pillars made of PDMS with and without stretching. After being seeded onto each substrate, cells were incubated with and without stretching for 6 h before being stained with rhodamine-phalloidin (RP) and 4′, 6-diamidino-2-phenylindole (DAPI). Scale bar=30 μm. Flat PDMS served as a control. (**b**) Percentage of PMEFs with long stable edges and long stress fibres and (**c**) cell area and cell aspect ratio on each substrate: flat PDMS (*n*=153 cells); unstretched soft pillars (*n*=159); soft pillars with 5% static stretching (*n*=105); soft pillars with 5% cyclic stretching at 0.1 Hz (*n*=175). Error bars, s.e.m. **P<0.01; Student’s *t*-test. Experiments were repeated at least three times.

As shown in Figs 1a–c and Supplementary Movie 1, primary mouse embryonic fibroblasts (PMEFs) plated on flat PDMS (polydimethylsiloxane; Young’s modulus (E)≈2 MPa)^14^ spread well with long, stable edges, stress fibres and a large spread area, as compared with those plated on soft pillars made of PDMS (2-μm height, 500-nm diameter, bending stiffness *k*=2.3 nN μm^−1^, Supporting Supplementary Material). These cellular responses were consistent with previous reports^11^,^12^,^25^ showing rigidity-dependent cell spreading and stress fibre formation on soft and rigid substrates such as gels and glass. To examine the effect of stretching on cell spreading and stress fibre formation, PMEFs were plated on soft submicron pillars coated with fibronectin and incubated with or without cyclic stretching by 5% in a microfabricated stretching device (Supplementary Figs 1 and 2). With no or a 5% static stretch, PMEFs on the pillars did not spread and form stress fibres on the soft pillars (Figs 1a–c; Supplementary Movie 1). In contrast, the cyclically stretched PMEFs (0.1 Hz) showed markedly increased cell spreading and stress fibre formation (Figs 1a–c), compared with the unstretched PMEFs. Thus, cyclic stretching enabled PMEFs to spread and form stress fibres even on soft substrates. In previous studies, it was also reported that the inhibition of Rho and Rho-kinase suppressed stress fibre formation on a rigid substrate, but fibres appeared after cyclic uniaxial stretch (10%, 1 Hz)^20^.

To test how general the phenomenon was, we performed similar experiments on primary human umbilical vein endothelial cells (HUVECs; Supplementary Fig. 3). Cells cultured on soft nanopillars without stretching for 6 h were less spread and with fewer long stress fibres compared with cells cultured with 5% stretching at 0.1 Hz. Thus, periodic stretching of soft pillars appears to provide a general activation signal for different cell types.

### Effect of frequency, magnitude and duration of the strain

**Figure 2:**
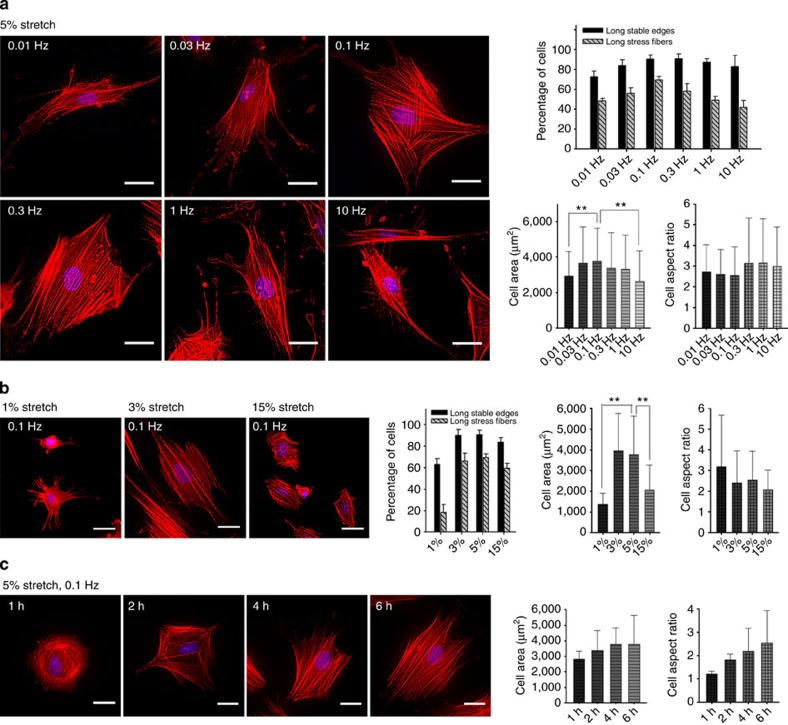
Effect of strain frequency and magnitude of cyclic stretching on cell spreading and stress fiber formation on soft pillars. (**a**) PMEFs on soft pillars with 5% cyclic stretching for 6 h at different frequencies: 0.01 Hz (*n*=126 cells); 0.03 Hz (*n*=92); 0.1 Hz (*n*=175); 0.3 Hz (*n*=171); 1 Hz (*n*=134); 10 Hz (*n*=174). (**b**) Cyclic stretch (0.1 Hz) for 6 h at various amplitudes: 1% (*n*=104 cells); 3% (*n*=132 cells); 5% (*n*=175); 15% (*n*=175). (**c**) 5% Cyclic stretching (0.1 Hz) for various periods of time: 1 h (*n*=113 cells); 2 h (*n*=95); 4 h (*n*=101 cells); 6 h (*n*=175). Error bars; s.e.m. **P<0.01; Student’s *t*-test. Cells were stained with RP and DAPI after stretching. Scale bar=30 μm. Experiments were repeated at least three times.

Cyclic stretching (5%) at frequencies ranging from 0.01 to 10 Hz caused a significant increase in spreading and stress fibre formation, with the optimum at 0.1 Hz (Fig. 2a). Similarly, 3% cyclic stretching at 0.1 Hz was equivalent to 5%, as shown in Fig. 2b. Even 1% stretch caused spreading, but the area was lower than with 3% and the axial ratio was higher. One explanation for the larger aspect ratio was the increase in displacement along the long axis. With a markedly higher strain of 15%, cyclic stretching at 0.1 Hz resulted in a significantly lower cell area (spreading) and cell aspect ratio (polarization) with no inhibition of stress fibre formation. Interestingly, 15% cyclic stretching of cells plated on flat PDMS also caused reduced cell area and cell aspect ratio with no inhibition of stress fibre formation (Supplementary Fig. 4). The excessive strain may have created very high stresses on the cytoskeleton that would cause it to retract. Thus, an excessive strain magnitude had a negative effect on cell spreading while a 1% strain stimulated the lengthening of the cells. Similar negative effects of large strain magnitude were reported previously^26^.

The effects of stretching duration on cell spreading and stress fibre formation were investigated. Cell spreading, polarization and stress fibre formation markedly increased when the stretching duration was increased from 1 to 4 h, and reached a plateau at 4 h, as shown in Fig. 2c. There was a lasting effect of cyclic stretching. When cells were cultured without further cyclic stretching for 18 h after the initial cyclic stretching at 0.1 Hz for various durations (1–4 h), many of the cells still remained well spread (Supplementary Fig. 5). After stretching for 4 h, >80% remained spread for 18 h with significant stress fibres (Supplementary Fig. 5). The level of spreading started to diminish at 24 h (decreased to 70%) and was largely reversed after 48 h where only 30% of the cells remained spread (Supplementary Fig. 6).

We looked for stress fibre and adhesion reversibility after prolonged stretching. In previous studies, inhibition of the Rho–ROCK–Myosin system by Y-27632 caused the loss stress fibres as on soft substrates but stress fibres returned on washing out the inhibitor^25^. If we stopped the cyclic stretching after 1 h, then the cell–matrix adhesions that formed during the cyclic stretching (Fig. 2c) were disassembled (Supplementary Fig. 5). However, if we stopped cyclic stretching after 4 h, stress fibres and focal adhesions persisted for 18 h (Supplementary Fig. 5).

### Relaxation time during each cycle affects stretching results

**Figure 3:**
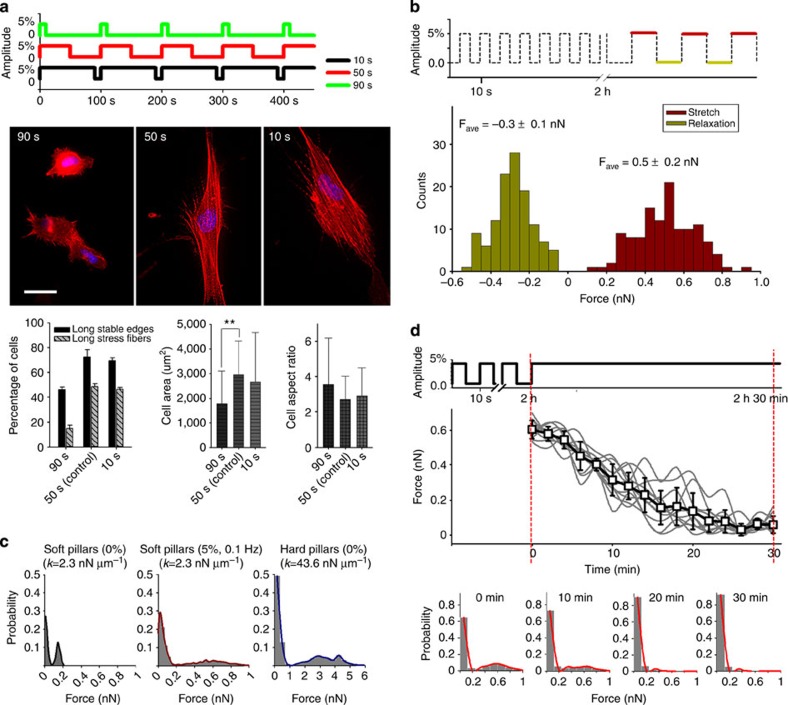
Effects of relaxation time of cyclic stretching on cell morphology and contractile responses of cells to cyclic stretch. (**a**) PMEFs on soft pillars with 5% cyclic stretching (0.01 Hz) for 6 h at different relaxation times: 10 s (*n*=95 cells); 50 s (*n*=126 cells); 90 s (*n*=114 cells). Cells were fixed 6 h after seeding and stained with RP and DAPI. Scale bar=30 μm. Error bars; s.e.m. **P<0.01; Student’s *t*-test. (**b**) Distributions of forces calculated from single stretch–relaxation event. Cells were first stretched for 2 h (5%, 0.1 Hz) after seeding and then displacements of pillars were calculated from single stretch–relaxation event (*n*=12 cells). Only the pillars near the cell edge were calculated. *F*_ave_ means average forces measured on stretch–relaxation. (**c**) Distributions of forces on soft or hard pillars (*n*>300 pillars). (**d**) Changes in forces over time on changing from 5% cyclic stretching after 2 h to 5% static stretching (*n*>300 pillars). Experiments were repeated at least three times and showed similar results.

Stretching (5%) at 0.01 Hz with 50 s of stretch and 50 s of relaxation (as a control) caused significant spreading but less than at 0.1 Hz (Figs 2a and 3a). To test if the time of relaxation versus stretching was important, cells were relaxed for 10 s and stretched for 90 s. This regime resulted in only slightly lower spread area and number of stress fibres than the 50/50 regime. However, when cells were relaxed for 90 s and stretched for 10 s in each cycle, there was a significant loss of cell spreading and stress fibre formation. We believe that this was an effect of the 90 s relaxation period and not the shortened stretch period, since in 50% stretch/relaxation cycles at 0.1 or 0.03 Hz, the stretch period is 5 or 17 s, respectively. Thus, the spreading is lost if the relaxation portion of the cycle is too long.

### Contractility studies in cyclic stretched cells

A potentially important aspect of the stretching response was the stimulation of contractility^6^,^12^,^16^. Contractile forces from cells were measured from the displacements of the pillars^14^,^16^. After cyclic stretching (5%, 0.1 Hz) of soft pillars for 2 h, we found that cells in the stretched state caused an inward displacement of the pillars near the cell edge by about 220 nm (significantly less than the 1.5–2 μm displacement of the pillars on opposite sides of the cells with a 5% stretch). Most surprising, however, in the relaxed state, cells caused outward displacements of the pillars by about 120 nm (Fig. 3b). The distribution of the outward forces (Supplementary Fig. 7 right panel and Supplementary Movie 2) showed that the greatest forces were 2–4μm behind the leading edge. This was the region of the endoplasmic portion of cytoplasm that may rigidify as a result of the periodic stretching and could possibly support compressive stress. Thus, the forces on the substrate indicated that the cells were stretching and relaxing only partially such that expansive and compressive stresses were applied in the stretched and relaxed states, respectively.

The displacement of stiff pillars (after 6 h without stretching) was similar to the displacement of soft pillars after cyclic stretching for 6 h (Fig. 3c). To understand why cyclic stretching had more remarkable effects in promoting cell spreading and stress fibre formation on soft substrates than static stretching, cells were held with 5% static stretching after 2 h of cyclic stretching (5%, 0.1 Hz), and pillar displacements were tracked over 30 min (Fig. 3d). Surprisingly, the number of pillars with large displacements (over 100 nm) markedly decreased over 30 min after stopping cyclic stretching (Fig. 3d). To determine if cyclic stretching altered the relaxation rate, we did a single stretch and followed pillars that were under high traction forces immediately after a stretch (0 min) and they relaxed over 10–20min. Thus, the relaxation time of pillar forces from a stretch was similar for cells before and after 2 h of cyclic stretching. A similar tendency of decreasing cellular contractility during static stretching was also observed in micron-scale pillars with comparable stiffness^16^. Thus, cyclic stimulation is required to prevent the gradual cellular adaptation to a relaxed configuration during the first 4 h.

### Cyclic stretch induces cell proliferation on soft substrates

**Figure 4:**
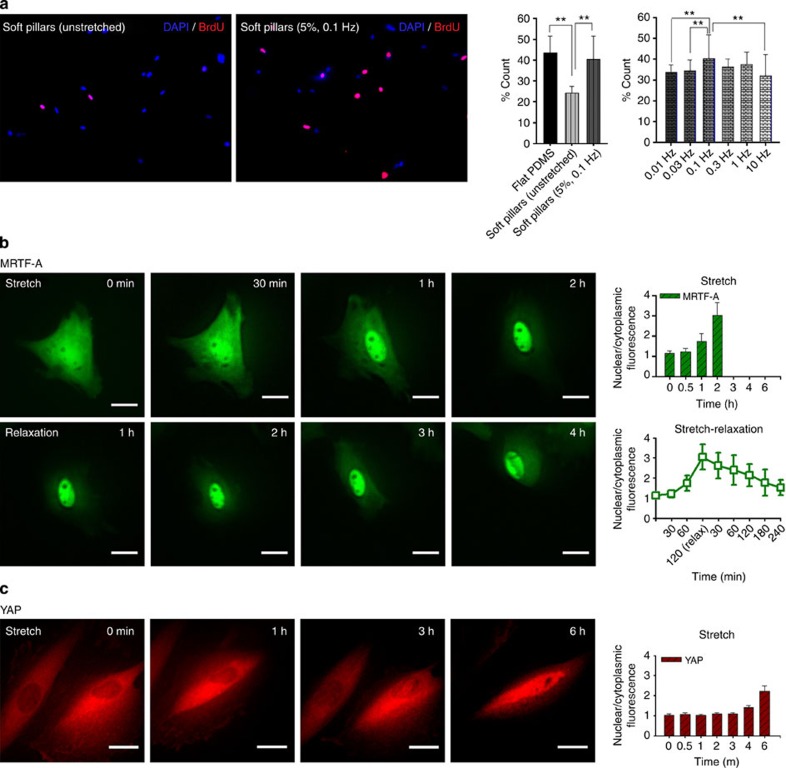
Effect of cyclic stretching on cell proliferation. (**a**) Percentage of cells costained with 5-bromo-2′-deoxyuridine (BrdU) and DAPI on different stretching types and frequencies: unstretched flat PDMS (*n*=140 cells), unstretched soft pillars (*n*=142), 5% cyclic stretched soft pillars at 0.1 Hz (*n*=74), 0.01 Hz (*n*=93), 0.03 Hz (*n*=66), 0.3 Hz (*n*=106), 1 Hz (*n*=59) and 10 Hz (*n*=79). Error bars; s.e.m. **P<0.01; Student’s *t*-test. Time-dependent translocation of (**b**) GFP–MRTF-A (green fluorescent protein tagged Myocardin-related transcription factor-A) and (**c**) YAP–GFP (green fluorescent protein tagged Yes-associated protein) by cyclic stretch (5%, 0.1 Hz). Plasmids were transient transfected into PMEFs through electroporation at least 24 h before imaging. Stretching was applied 2 h after the seeding of cells in the chamber. Over 20 cells were analysed for each protein. Experiments were repeated at least three times and showed similar results. Scale bar=20 μm.

With 5% cyclic stretching at 0.1 Hz, cell proliferation was greatly increased to a level similar to that observed with cells grown on hard substrates (Fig. 4a). The stimulation of cell proliferation changed with frequency of stretch in a manner similar to the stimulation of cell spreading. The largest increase in growth was observed at 0.1 Hz, and the lowest was observed at the two ends of the tested frequencies, 0.01 and 10 Hz. Thus, cyclic stretching can induce cell proliferation as well as spreading on soft substrates.

Because cells were proliferating on soft substrates and had an increased density of stress fibres, the cells may have been responding to the movement of actin motility related factors to the nucleus such as MRTF-A or YAP^27^,^28^,^29^,^30^. When we followed the distribution of MRTF-A in cells on soft surfaces, there was no accumulation in the nucleus until the substrate was stretched cyclically (a plateau was reached after 2 h of cyclic stretching, Fig. 4b). Further, when cyclic stretching was stopped at 2 h, the MRTF-A moved back into the cytoplasm. When we increased the cyclic stretching time to 8 h, there was continued movement of MRTF-A to the nucleus and MRTF-A tended to accumulate in localized regions of the nucleus (Supplementary Fig. 8). After cessation of the stretching at 8 h, MRTF-A moved into the nucleus for an additional 2 h and then started to slowly move out from nucleus to cytoplasm but at a slow rate. In the case of YAP, the movement to the nucleus was much slower and only after 6 h was there a significant accumulation of YAP in the nucleus (Fig. 4c). Thus both MRTF-A and YAP tend to accumulate in the nuclear after stretch.

To further investigate if MRTF-A and YAP were important for spreading and proliferation, the levels of MRTF-A and YAP were knocked down by siRNA. Depletion of MRTF-A also affected expression of YAP; however, expression of MRTF-A was not altered by the siRNA depletion of YAP (see Supplementary Fig. 9a). On rigid glass substrates, depletion of MRTF-A prevented translocation of endogenous YAP to the nucleus but had no effect on cell spreading or formation of stress fibres (Supplementary Fig. 9b). Interestingly, on soft nanopillars, cyclic stretching (5%, 0.1 Hz) induced accumulation of YAP surrounding the nucleus and cells did not spread and/or form stress fibres (Supplementary Fig. 9b). With increasing time of stretching, the MRTF-A siRNA cells lost contractility and rounded up (cells behaved similarly with expression of YAP–GFP or GFP, Supplementary Fig. 10a). YAP–GFP accumulated around the nucleus, but not in it; whereas control GFP protein did not accumulate around nucleus in the MRTF-A siRNA cells following cyclic stretch and only celluar contractility was affected (Supplementary Fig. 10a).

Following depletion of YAP with siRNA, the nuclear/cytoplasm ratio of MRTF-A was near 1 in most cells on a rigid glass substrate and cells spread well (Supplementary Fig. 9b). However, on soft nanopillars, cells did not spread and form stress fibres with cyclic stretch (5%, 0.1 Hz). Similarly, time-dependent fluorescence images of PEMF cells cotransfected with YAP siRNA and GFP–MRTF-A plasmid (Supplementary Fig. 10b) also showed that cells lost contractility and rounded up within 30 min after cyclic stretch was applied (at later times, blebbing indicated that the cells were likely to apoptose). In comparison, control siRNA did not affect either MRTF-A translocation or cyclic stretch induced cell spreading (Supplementary Fig. 10c). Thus, depletion of either MRTF-A or YAP was sufficient to inhibit stretch-induced spreading and movement of the other protein to the nucleus. We suggest that cyclic stretch-induced movements of both MRTF-A and YAP to the nucleus are needed for stimulation of spreading and proliferation on soft surfaces.

Prolonged stretching for >4 h causes marked changes in the cell organization. This is the first documentation of such an alteration in cell responsiveness to the substrate. Since all of these changes require the presence of MRTF-A and YAP, it is likely that new protein expression is required for this stabilization of stress fibres and adhesions on soft surfaces. When stabilized cells are released from the pillars and placed on fresh pillars, they do not spread, indicating that the process of early adhesion formation is not altered in the cells. We hypothesize that the stabilization occurs through the modifications of the adhesions and/or stress fibres to enable continued contractility on soft substrates.

Distortions of tissue matrices by 1–5% are very common even in non-muscle tissues and are part of normal activity such as athletics, walking or even breathing. For tissues with otherwise soft matrices such as skin and relaxed muscle, physical activity could provide a major stimulus for cell growth irrespective of hormones or other factors. Fibroblasts that have been grown on hard, tissue plastic surfaces are dependent on either a rigid substrate, where cytoskeleton-generated forces will test matrix rigidity or periodic matrix stretch–relaxation cycles that will modulate forces on integrins. Since both types of matrix forces are transmitted through the cytoskeleton-adhesion interface, they could share a common activation pathway. The steps in the process appear to involve the serum response pathway that depends on actin filament assembly either downstream of external matrix-generated or cell-generated forces. However, mechanical stimulation of the cells by cyclic stretching is essential for growth on soft surfaces.

## Methods

### Pillar fabrication

PDMS (polydimethyl siloxane; 10:1=PDMS prepolymer:curing agent, w/w) (Sylgard 184, Dow Corning) was spin-coated over a silicon mould^13^ at 1,000 rpm for 1 min to obtain a 60-μm thick membrane. After 3 h of curing at 80 °C, the membrane was peeled off from the mould and served as a flexible cell culture substrate in the stretching device (Supplementary Fig. 1c). Pillar arrays were 500 nm in diameter and 2 μm in height, with center-to-center separation of 1 μm to maintain constant areal density. The pillar bending stiffness was calculated according to Euler–Bernoulli beam theory^31^.

### Stretching device fabrication

The design of the stretching device is depicted in Supplementary Figs 1a and 1b. The device is similar to the stretching system introduced by Moraes *et al*.^32^, and consists of multiple layers of patterned PDMS fabricated by multilayer soft lithography (Supplementary Fig. 1c)^16^,^33^. Each stretching unit consists of a 3-mm wide culture chamber with a PDMS membrane layer underneath and a post loading layer (2-mm wide and 100-μm high) of PDMS (5:1=PDMS prepolymer:curing agent, w/w) suspended over the third actuation cavity layer. Positive pressure (Gas Generator, Fisher) was applied to this cavity to push the loading post upwards, which deforms the flexible PDMS membrane (Supplementary Fig. 1b). A lubricant (90% glycerol) was added between the loading post and the culture membrane to prevent adhesion between the components. For cyclic stretching, different frequencies (0.1–10 Hz) were generated using a commercial gas regulator (Fluidigm, South San Francisco, CA, USA). To generate even lower frequencies of 0.01 and 0.03 Hz, an additional asymmetrical recycler timer relay (ABB, Cary, NC, USA) was incorporated into the gas regulator, bypassing the triggering mechanism.

### Device characterization

Surface strains on the device were first characterized by measuring changes of center-to-center distance of 2 μm pillar arrays^5^. Various pressures were applied to characterize the magnitude of the strain generated at each pressure through the changes of center-to-center distance (Supplementary Fig. 2a). As shown in Supplementary Fig. 2b, the surface strain directly on the nanopillar surface was also examined. First, the pillar arrays in the stretching devices were stamped with fluorescent fibronectin-Cy3 (GE Healthcare). Then, the tips of the pillars were imaged using fluorescence microscopy (Olympus Live TIRF) with or without stretching, and the centroids of the pillar tips were calculated using a custom algorithm in Image J (NIH). Since the device was expected to generate equibiaxial stretching, circumferential strain parameters were also plotted with regards to changes in circumferential bead location (Supplementary Fig. 2b). Moreover, to determine the consistency of strain over time under either cyclic or static stretching, pressure was applied either statically or at 0.1 Hz for up to 6 h. Every hour, strain magnitudes were calculated from 10 randomly selected points, and their average values were plotted and compared with each other (Supplementary Fig. 2b).

### Cell culture and staining for fluorescent microscopy

PMEFs (provided by Dr G.V.Shivashankar in National University of Singapore) were cultured in DMEM (Dulbecco's Modified Eagle Medium; Gibco, Life Technologies) supplemented with 10% FBS (foetal bovine serum; Gibco, Life Technologies) and maintained in an incubator at 37 °C with 5% CO_2_. The surface of the stretching device was functionalized with 10 μg ml^−1^ of fibronectin overnight at 4 °C. Stretching was applied right after the seeding of cells in the chamber. For actin staining, cells were washed with PBS (phosphate-buffered saline), fixed in 0.25% glutaraldehyde and 0.05% Triton X-100 for 1 min, followed by 1% glutaraldehyde for 10 min, and post fixed for 45 min in NaBH4, all in PBS. Cells were incubated with rhodamine–phalloidin (RP; 200 units in DMSO, R415, Molecular Probes, Life Technologies) diluted at 1:1000 in PBS for 30 min. DNA was stained with 4′, 6-diamidino-2-phenylindole (DAPI; 0.2 mg ml^−1^ in PBS, D9542, Sigma-Aldrich Co.) at room temperature for 5 min. Images were acquired on a Delta Vision System (Applied Precision) centred on an Olympus IX70 microscope and equipped with a CoolSNAP *HQ*^2^ CCD camera (Photometrics, Tucson, AZ, USA). For BrdU staining, after 2 h of stretching, BrdU labelling reagent was added to the culture media at a final concentration of 10 μM, and cells were incubated at 37 °C for 6 h with stretching. After cells were fixed with 4% paraformaldehyde for 30 min and treated for 30 min in 2 N HCl, they were incubated with anti-BrdU (5-bromo-2′-deoxyuridine) antibody conjugated with fluorescein according to the manufacturer’s instructions (Roche). Images of BrdU-incorporated cells were acquired on an EVOS digital fluorescence microscope (Fisher Scientific). For live imaging, GFP–MRTF-A or YAP–GFP plasmids were transient transfected into PMEFs through electroporation using Neon Transfection System (Life Technologies) at least 24 h before imaging. Stretching was applied 2 h after the seeding of cells in the chamber.

### Traction force measurement

To measure the forces on the cells, the pillar arrays in the stretching devices were stamped with fluorescent fibronectin-Cy3 (GE Healthcare). The tips of the pillars were imaged using fluorescence microscopy (Olympus Live TIRF) and the centroids of the pillar tips were calculated using a custom algorithm in Image J (NIH). The deviation of the tips of the pillars from the calculated array coordinates was calculated and taken as the displacement of the pillar tip.

## Author contributions

Y.C. and F.M.H. performed the experiments and analysed the data. M.S. designed the experiments and Y.C., S.P. and M.S. wrote the paper. B.Y. contributed technical assistance with transfection, Q.P. contributed technical assistance with siRNA transfection and immunoblotting and K.L. contributed technical assistance with BrdU staining.

## Additional information

**How to cite this article:** Cui, Y. *et al*. Cyclic stretching of soft substrates induces spreading and growth. *Nat. Commun.* 6:6333 doi: 10.1038/ncomms7333 (2015).

## Supplementary Material

Supplementary Information Supplementary Figures 1-10 and Supplementary Methods.

Supplementary Movie 1 Primary Mouse Embryonic Fibroblasts (PMEFs) plated on flat PDMS and on soft pillars made of PDMS (2-μm height, 500-nm diameter, bending stiffness k = 2.3 nN/μm).

Supplementary Movie 2 The movie shows the movement of pillars during the relaxation step after 50s' stretch. Images were taken from one cycle of stretch and relaxation at 0.01Hz (stretched for 50s and relaxed for 50s) at a framing rate of 1 frame/s. Scale bar = 100nm displacement.
