# Supplementary material for: Cyclic stretching of soft substrates induces spreading and growth
Source: Nat Commun. 2015 Feb 23;6:6333. doi: 10.1038/ncomms7333 (PMC4346610; doi:10.1038/ncomms7333)
Supplement: Supplementary Information — Supplementary Figures 1-10 and Supplementary Methods. [file ncomms7333-s1.pdf]

Cyclic Stretching of Soft Substrates Induces Spreading and Growth

Yidan Cui, Feroz M. Hameed, Bo Yang, Kyunghee Lee, Catherine Qiurong Pan, Sungsu Park, Michael Sheetz

Supplementary Information

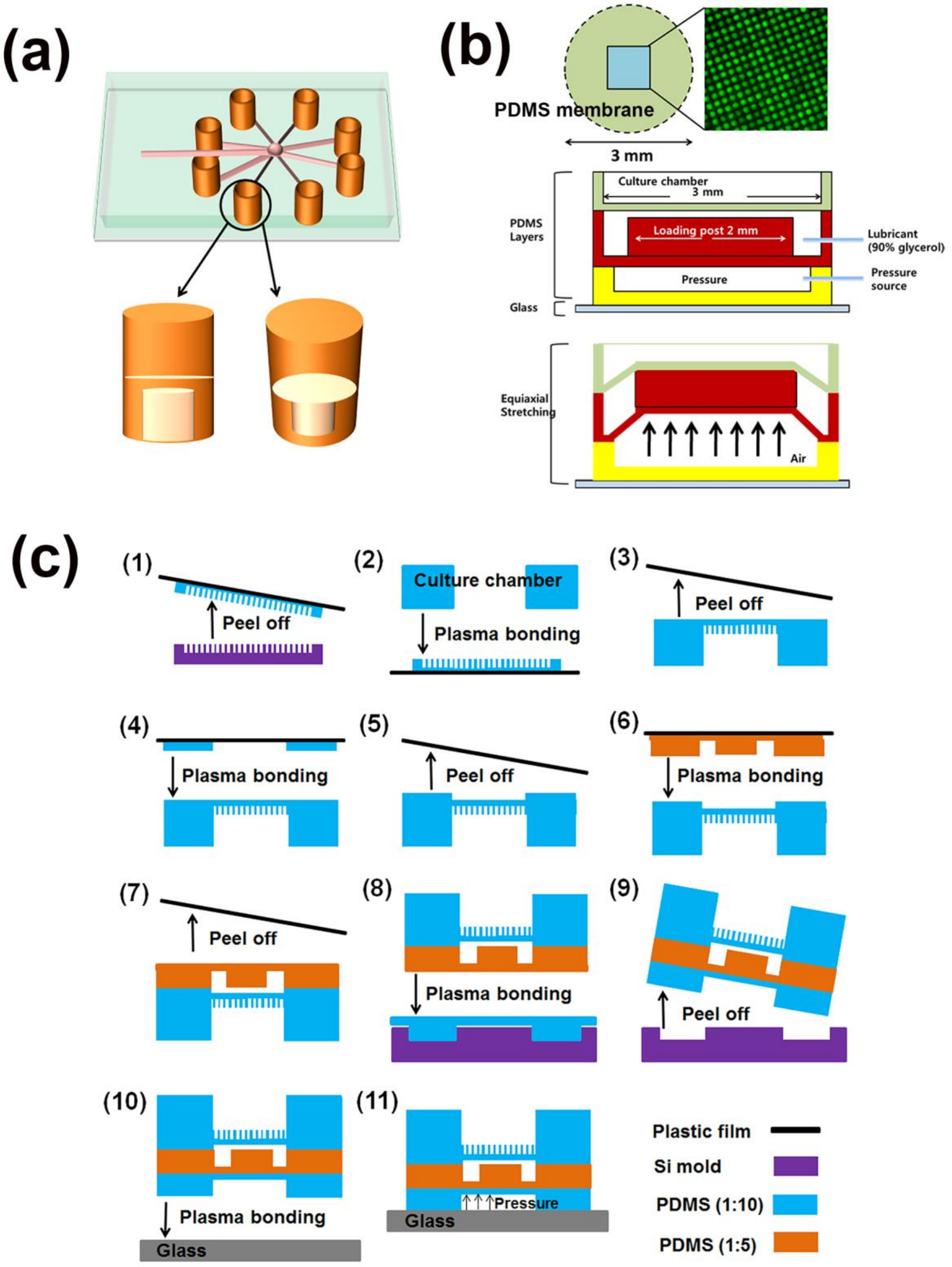

Supplementary Figure 1. Design and fabrication. (a) Design of the stretching device. **(b)** Cross-sectional design of each stretching unit. Each stretching unit consists of a 3-mm-wide culture chamber with a PDMS (polydimethylsiloxane) membrane layer underneath and a post loading layer (2-mm wide and 100-µm high) of PDMS suspended over the third actuation cavity layer. Positive pressure was applied to this cavity to push the loading post upwards, which deforms the flexible PDMS membrane. **(c)** Fabrication process of the stretching device.

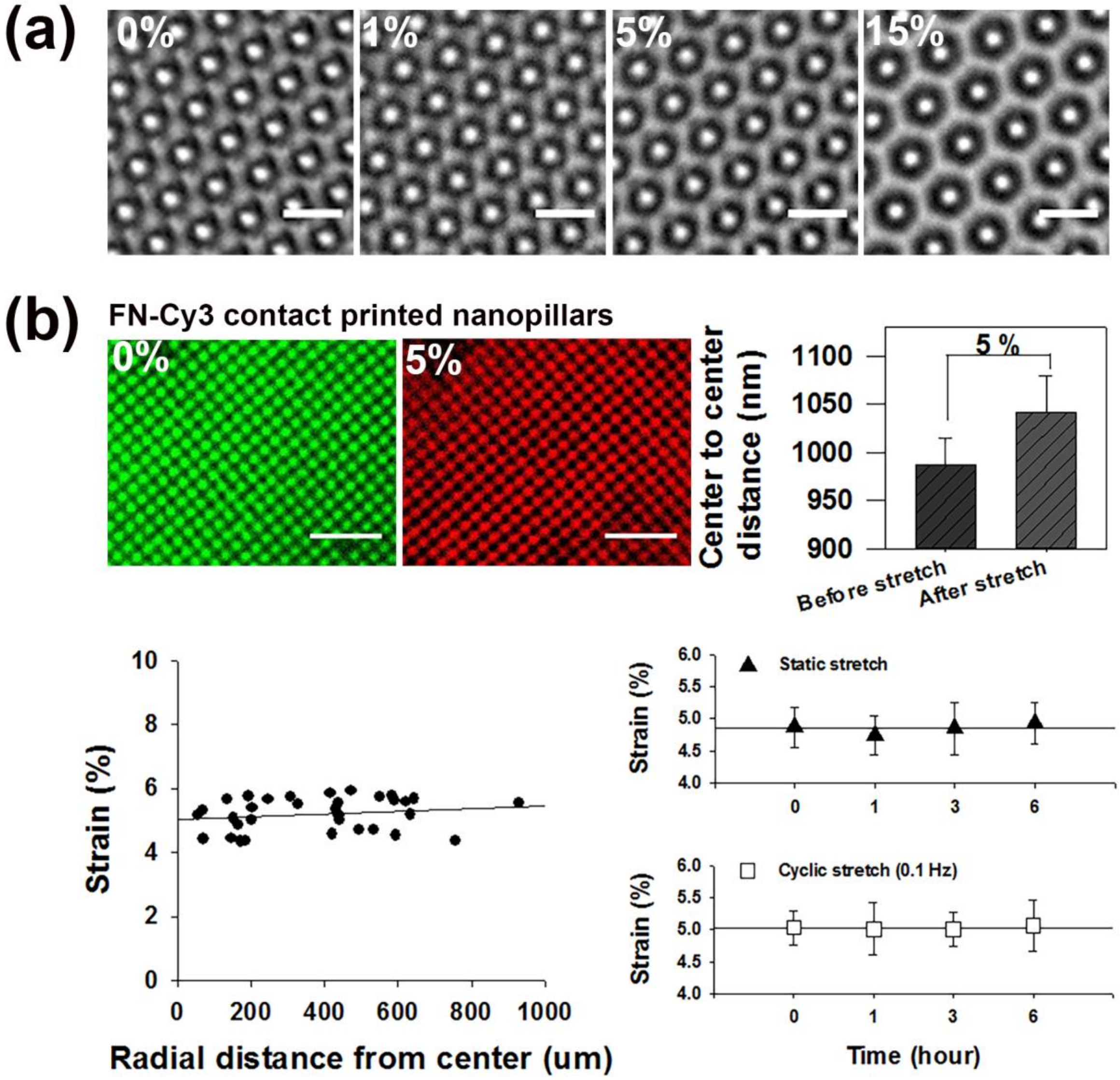

Supplementary Figure 2. Device characterization. (a) Changes of center to center distance (2 µm pillar arrays) by 1%, 5% and 15 % stretch. Scale bar = 5 um. **(b)** Changes of center-to-center distance after 5% cyclic stretching for 6 hrs (n > 300 pillars). Surface of the pillar array was contact printed with fluorescent fibronectin-Cy3. Scale bar = 5 um. Characterization of strains on device surfaces. Circumferential strain parameters were plotted (Left) and consistency of strains over time under either cyclic or static stretching pressure was also measured (Right).

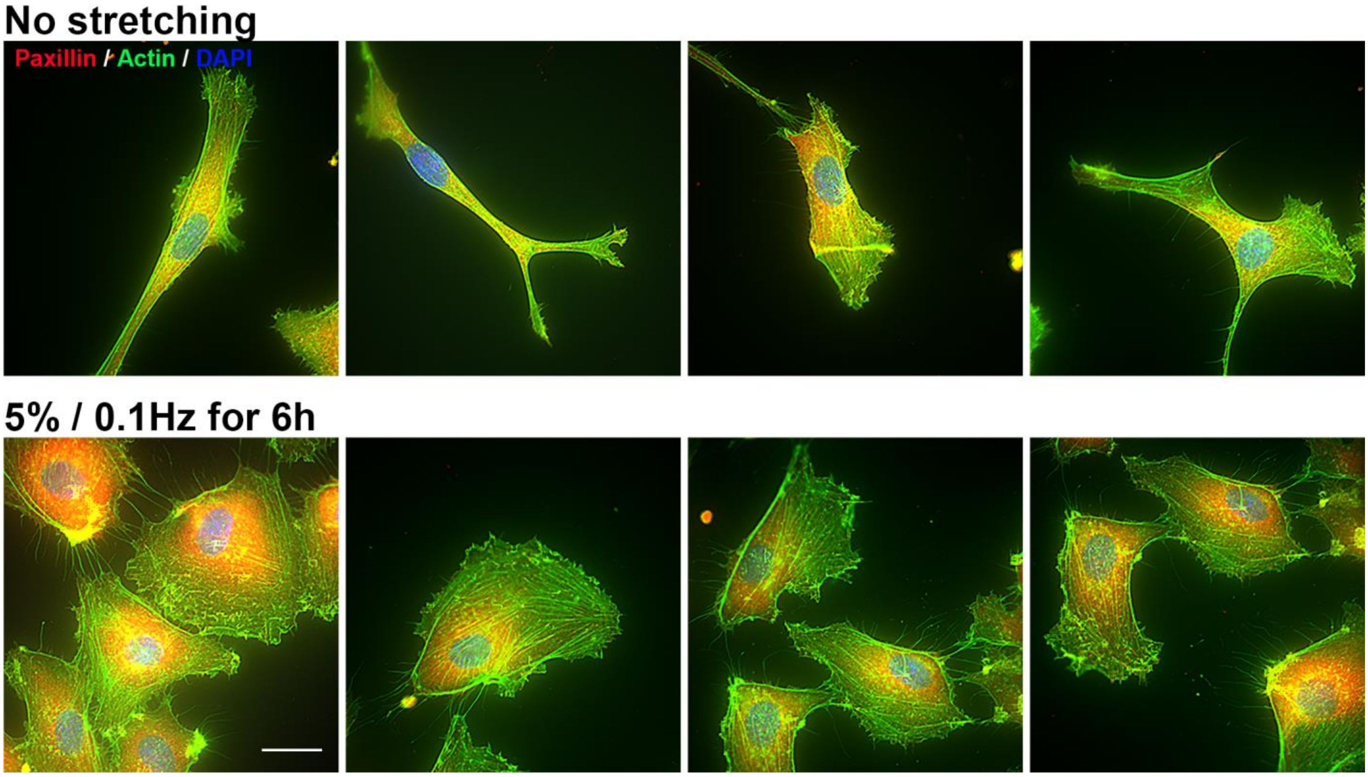

Supplementary Figure 3. Test the effect of cyclic stretching on cell spreading and stress fiber formation of another cell type. Representative images of primary human umbilical vein endothelial cells (HUVECs) on soft pillars made of PDMS with and without stretching. After being seeded onto substrate, cells were incubated with and without stretching for 6 hrs before being stained with fluorescein isothiocyanate labeled phalloidin, anti-paxillin antibody and 4', 6-diamidino-2-phenylindole (DAPI). Scale bar = 20 μm. Experiments were repeated three times and showed similar results.

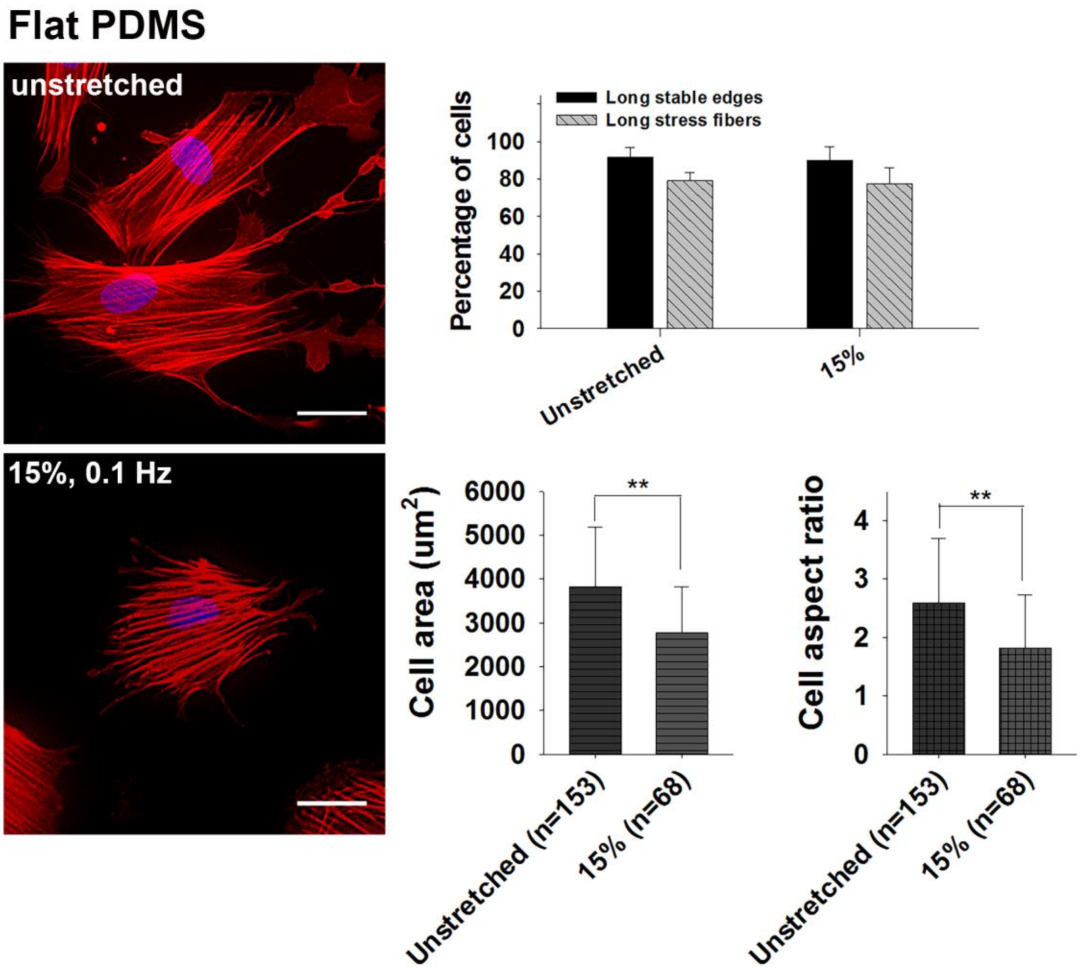

Supplementary Figure 4. Effect of large magnitude (15%) of cyclic stretching on cell spreading and stress fiber formation on a flat PDMS surface. Percentage of PMEFs with long stable edges and long stress fibers and cell area and cell aspect ratio on each substrate: flat PDMS (n = 153 cells); soft pillars with 15% cyclic stretching at 0.1 Hz (n = 68). Cells were fixed after 6 hrs of stretching and then stained with rhodamine-phalloidin and DAPI. Scale bar = 30 µm. Error bars, s.e.m. ** $P < 0.01$; Student's *t*-test. Experiments were repeated at least three times.

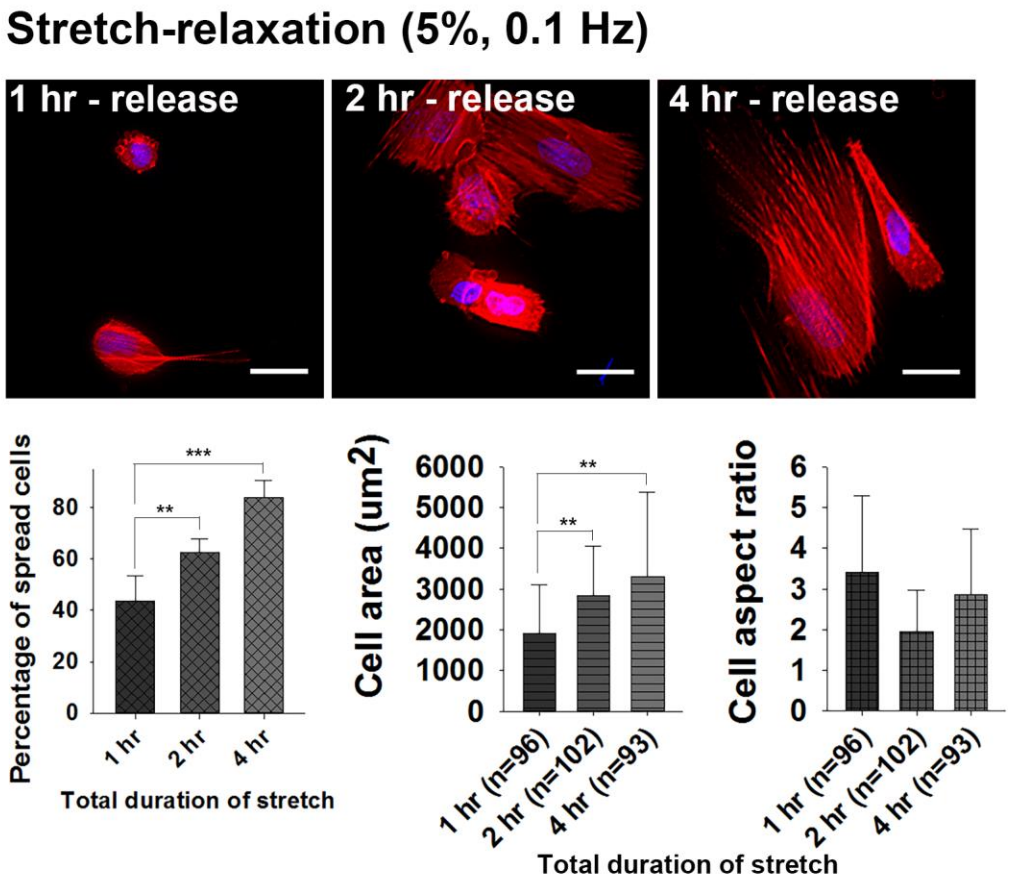

Supplementary Figure 5. Effect of total duration of cyclic stretching and subsequent relaxation time on cell spreading and stress fiber formation on soft pillars. Percentage of spread PMEFs and cell area and cell aspect ratio of PMEFs. Cyclic stretching was stopped 1 hr (n = 96 cells), 2 hrs (n = 102) and 4 hrs (n = 93) after seeding and cells were then statically cultured for 18 hrs before being stained with rhodamine-phalloidin and DAPI. Scale bar = 30 μm. Error bars: s.e.m. ** $P < 0.01$, *** $P < 0.001$; Student's *t*-test. Experiments were repeated at least three times.

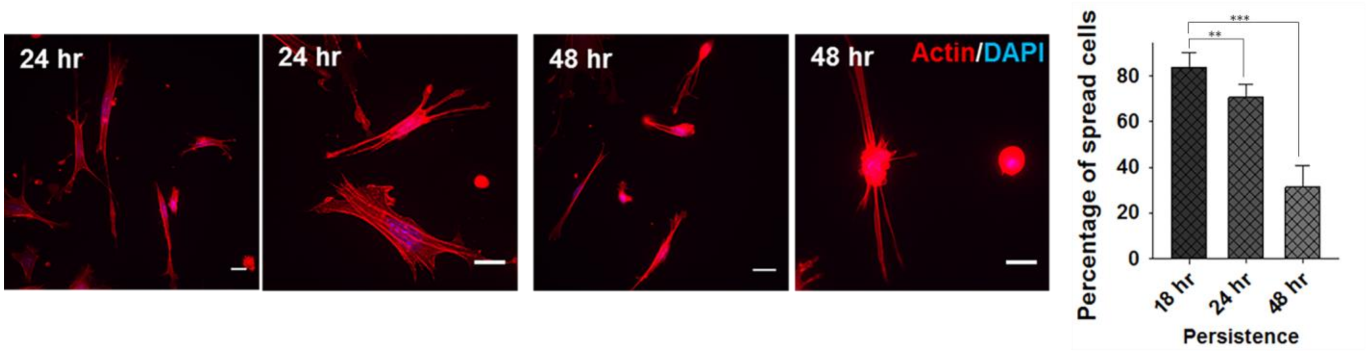

Supplementary Figure 6. Persistence of spreading. Cells were cultured without further cyclic stretching for 24 hrs (n = 81) or 48 hrs (n = 79) after the initial cyclic stretching at 0.1 Hz for 4 hrs. Then cells were fixed and stained with rhodamine-phalloidin and DAPI. Scale bar = 30 µm. Error bars: s.e.m. ** $P < 0.01$, *** $P < 0.001$; Student's *t*-test. Experiments were repeated three times and showed similar results.

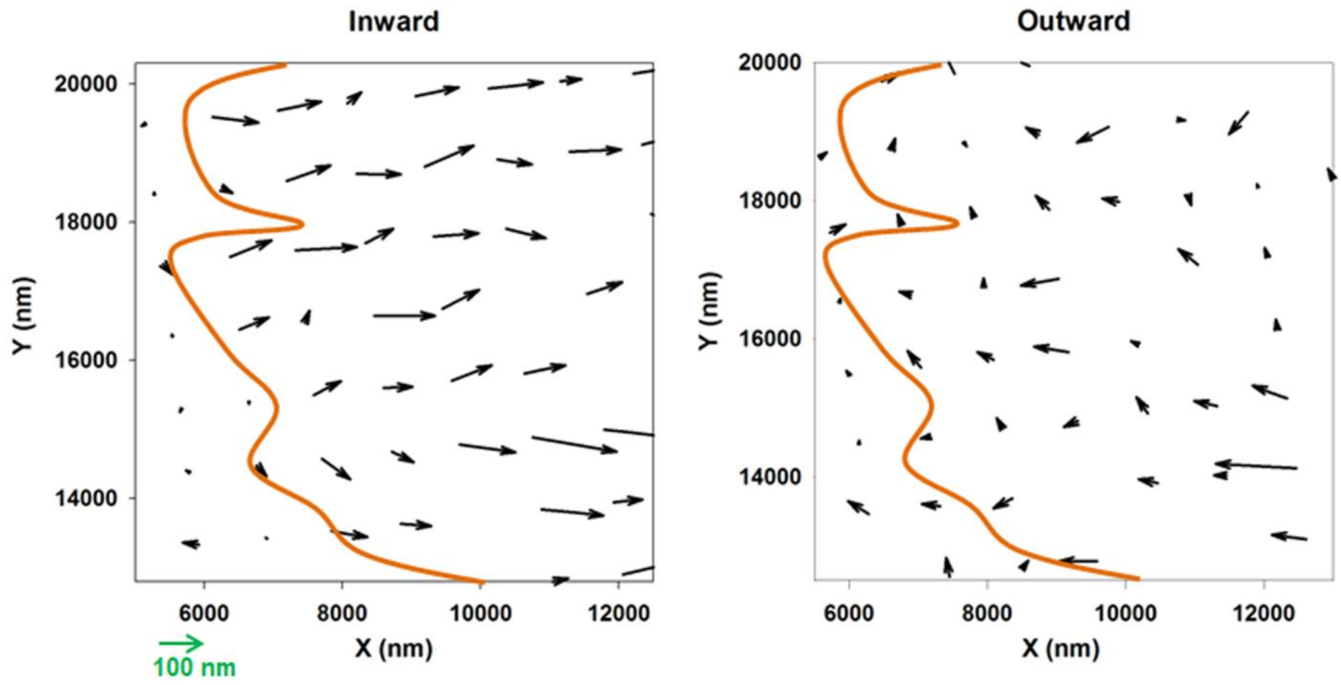

Supplementary Figure 7. Displacement of pillar displacements near the edge of a single cell upon both stretch and relaxation. The orange line represents the approximate cell boundary. The green arrow shown at the bottom of the image corresponds to a 100 nm displacement.

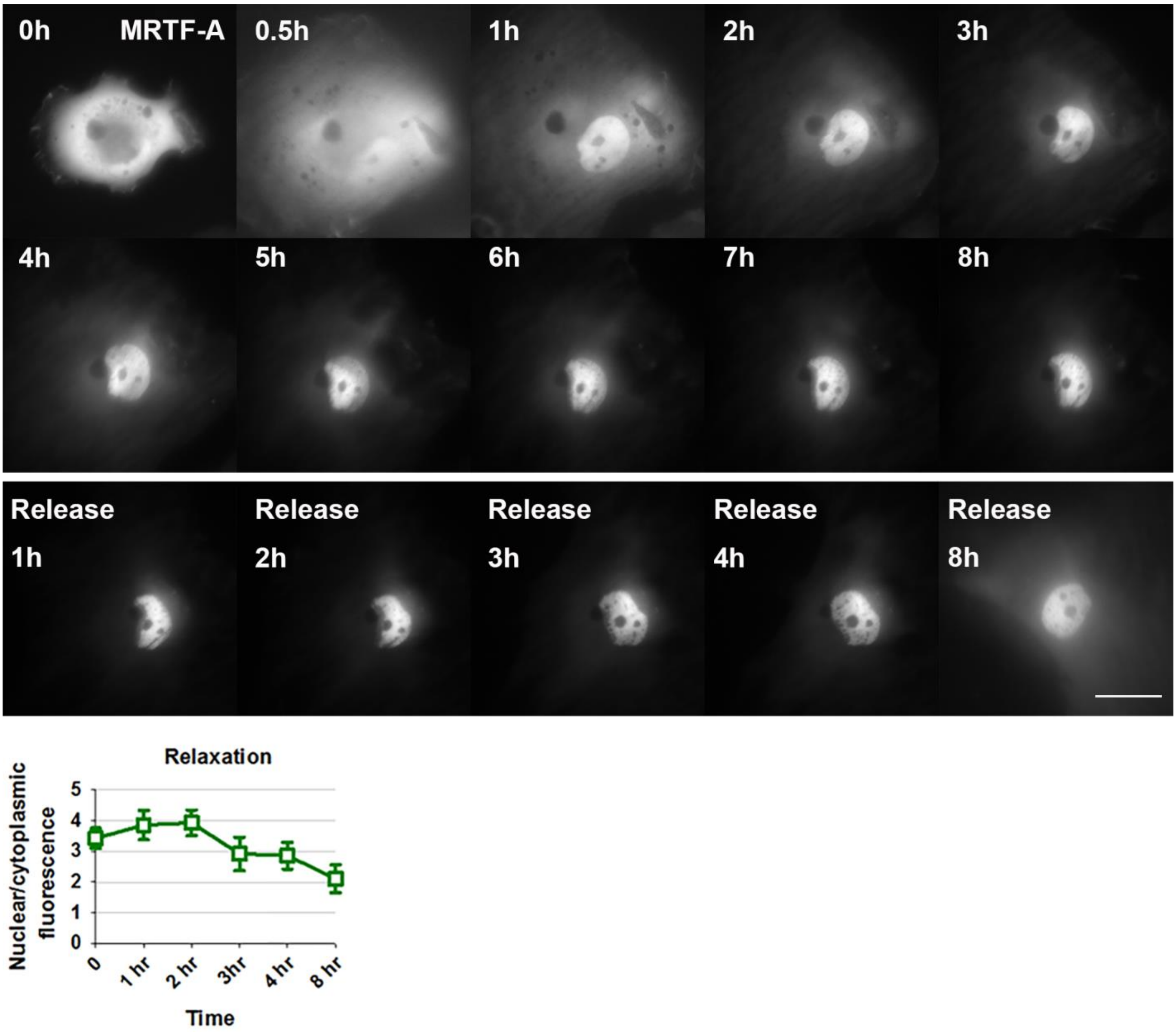

Supplementary Figure 8. Time-dependent translocation of GFP-MRTF-A (green fluorescent protein tagged Myocardin-related transcription factor-A) to the nucleus by cyclic stretch (5%, 0.1 Hz). Plasmids were transiently transfected into PMEFs through electroporation at least 24 hrs before imaging. Stretching was applied 2 hrs after the seeding of cells in the chamber. Over 20 cells were analyzed. Experiments were repeated three times and showed similar results. Scale bar = 30 µm.

1 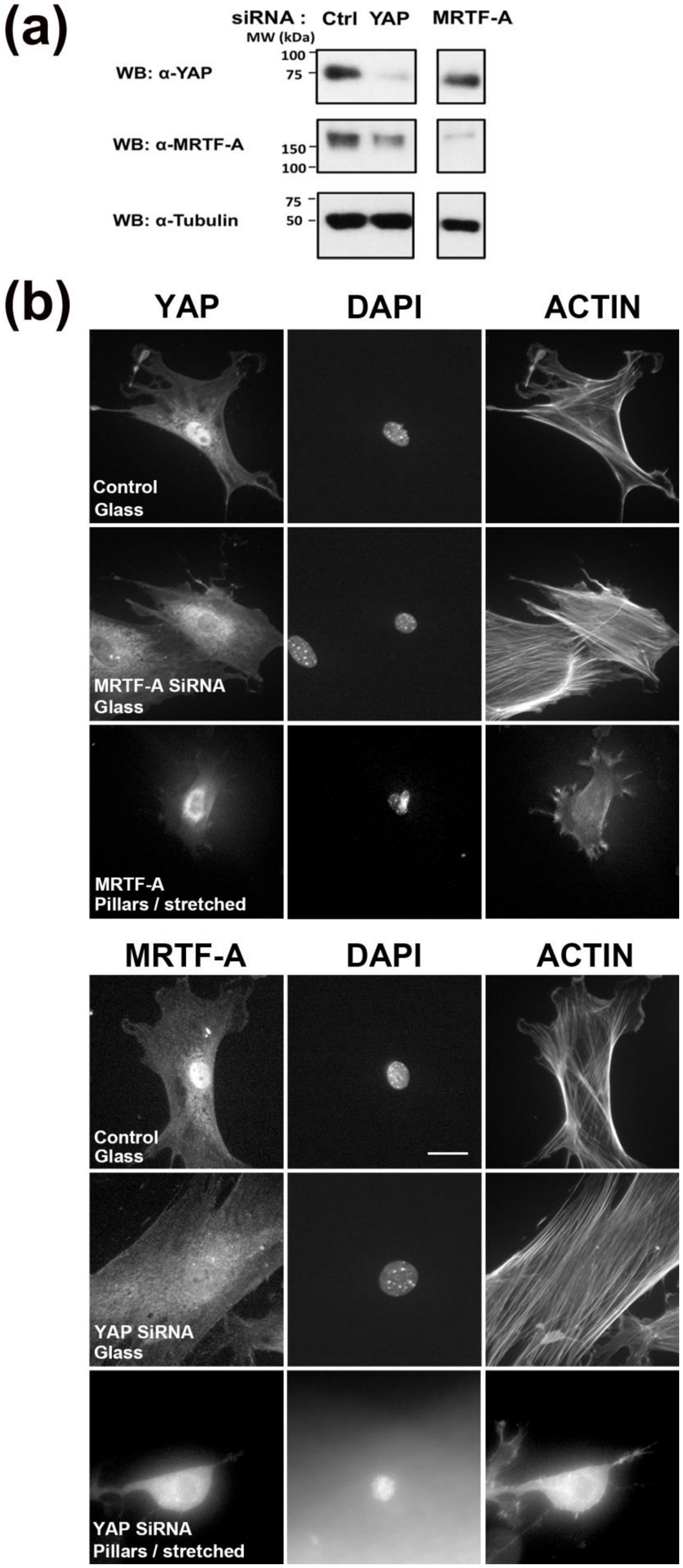

(a)
siRNA : Ctrl YAP MRTF-A
MW (kDa)
WB: α-YAP
100
75
WB: α-MRTF-A
150
100
WB: α-Tubulin
75
50
(b)
YAP
DAPI
ACTIN
Control
Glass
MRTF-A SiRNA
Glass
MRTF-A
Pillars / stretched
MRTF-A
DAPI
ACTIN
Control
Glass
YAP SiRNA
Glass
YAP SiRNA
Pillars / stretched

Supplementary Figure 9. Knockdown experiments. (a) Immunoblotting from PMEF cells transfected with the indicated siRNAs. **(b)** Immunofluorescence images of YAP (Yes-associated protein), MRTF-A, DAPI and actin in PMEF cells transfected with the indicated siRNAs (control indicates no siRNA treatment). For immunostaining, cells were trypsinized and seeded either on stretch device or glass coverslips coated with fibronectin. Cells were stretched (5% at 0.1 Hz) for 6 hours (stretching was applied 2 hrs after seeding of cells in the chamber) on stretch device, or static cultured for 8 hours on glass coverslips. Then cells were fixed and stained with MRTF-A and YAP antibodies, rhodamine-phalloidin and DAPI. Experiments were repeated three times and showed similar results. Scale bar = 30 µm.

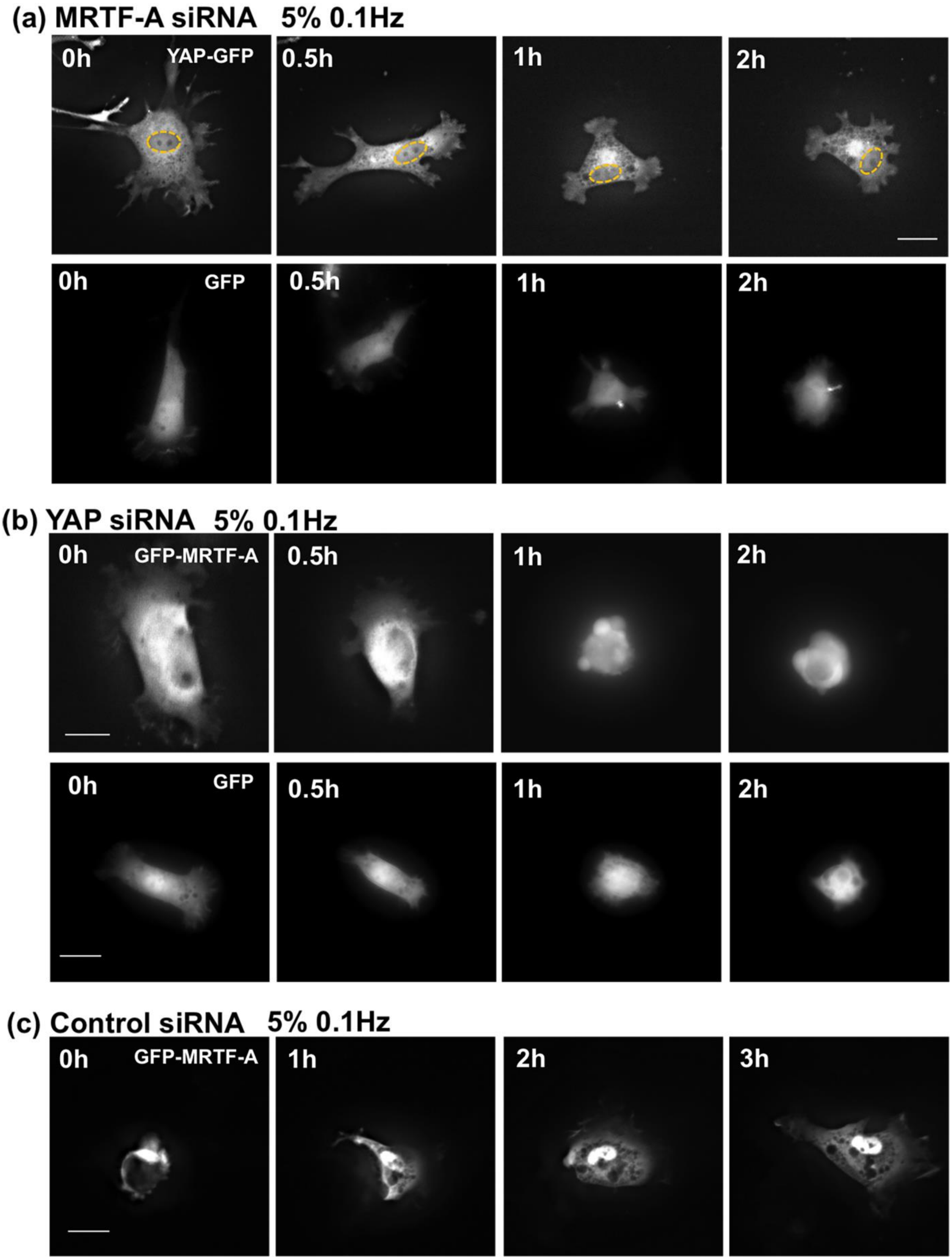
(a) MRTF-A siRNA 5% 0.1Hz
0h
YAP-GFP
0.5h
1h
2h
0h
GFP
0.5h
1h
2h
(b) YAP siRNA 5% 0.1Hz
0h
GFP-MRTF-A
0.5h
1h
2h
0h
GFP
0.5h
1h
2h
(c) Control siRNA 5% 0.1Hz
0h
GFP-MRTF-A
1h
2h
3h

Supplementary Figure 10. Time dependent imaging of knockdown cells. (a) Time-dependent fluorescence images of PMEF cells co-transfected with the MRTF-A siRNA and YAP-GFP (green fluorescent protein tagged Yes-associated protein) plasmid. Yellow circles in the figure indicate cell nuclear. **(b)** Time-dependent fluorescence images of PMEF cells co-transfected with the YAP siRNA and MRTF-A-GFP plasmid. **(c)** Time-dependent fluorescence images of PMEF cells co-transfected with the control siRNA and MRTF-A-GFP plasmid. Stretching was applied 2 hrs after seeding of cells in the chamber. At least 10 cells were analyzed for each condition. Experiments were repeated three times and showed similar results. Scale bar = 20 μm.

Supplementary Methods.

Cell culture and staining for fluorescent microscopy. HUVECs were cultured in phenol red-free Medium 200 (Medium 200PRF; M-200PRF-500, Life Technologies) supplemented with Low Serum Growth Supplement (LSGS; S-003-10, Life Technologies) and maintained in an incubator at 37 °C with 5% CO_2. The surface of the stretching device was functionalized with 10 μg/ml of fibronectin overnight at 4 °C. Stretching was applied right after the seeding of cells in the chamber. For actin and paxillin staining, cells were washed with PBS (phosphate-buffered saline), fixed in 4% paraformaldehyde for 20 min and permeabilized in 0.5% Triton X-100 for 10 min, followed by blocking with 3% bovine serum albumin for 30 min in room temperature, all in PBS. Cells were incubated with Anti-Paxillin antibody (Mouse, ab3127, Abcam) diluted at 1:500 in PBS for overnight at 4 °C. Then cells were washed three times with 0.05% Triton X-100 in PBS and incubated with Alexa Fluor 594 Goat Anti-Mouse IgG Antibody (A-11005, Molecular Probes) and fluorescein isothiocyanate labeled phalloidin (P5282; Sigma-Aldrich Co) diluted at 1:1000 in PBS for 30 min. DNA was stained with 4', 6-diamidino-2-phenylindole (DAPI; 0.2 mg/ml in PBS, D9542, Sigma-Chemical Co.) at room temperature for 5 min. Images were acquired on a Delta Vision System (Applied Precision) centered on an Olympus IX70 microscope and equipped with a CoolSNAP HQ^2 CCD camera (Photometrics, Tucson, AZ).

siRNA transfections. siRNA transfections were done with DharmaFECT™ (Dharmacon™) in antibiotics-free DMEM according to manufacturer's instructions. The siRNA sequences of the SMARTpool® (Dharmacon™) mouse YAP are 5'- CCGAAAUCUUGGACGUGGA - 3', 5'- GAAUAAAGGAUGGCGUCUU - 3', 5'- UCUUAAAUCACAACGAUCA - 3' and 5'- AAGGAGAGACUGCGGUUGA - 3'. The siRNA sequences of the SMARTpool® mouse MRTF-A are 5'- GGACCGAGGACUAUUUGAA- 3', 5'- GCUGCGUCCUGCUGUCUAA - 3', 5'- GGUCAGCUCUUGUAACAGC - 3' and 5'- GCACAUGGAUGAUCUGUUU - 3'.

Western blotting. Cells were lysed with RIPA buffer [150 mM NaCl, 50 mM Tris-HCl pH 7.3, 0.25 mM EDTA, 1% (w/v) sodium deoxycholate, 1% (v/v) Triton X-100, 50 mM NaF, 5 mM sodium orthovanadate, protease

inhibitors (Roche Applied Science)]. Samples were run in SDS-PAGE gels followed by western blot analyses using the Pierce Pico ECL (Thermo Scientific). Polyclonal anti-YAP (Rabbit, provided by Prof. Marius Sudol, National University of Singapore), polyclonal goat anti-MRTF-A (Santa Gruz Biotechnology) and monoclonal anti-α tubulin (Sigma-Aldrich Co) were used.

Immunofluorescence imaging of knockdown cells. Cells were washed with PBS (phosphate-buffered saline), fixed in 4% paraformaldehyde for 20 min and permeabilized in 0.5% Triton X-100 for 10 min, followed by blocking with 3% bovine serum albumin for 30 min in room temperature, all in PBS. Cells were incubated with Anti-YAP (Rabbit, provided by Prof. Marius Sudol, National University of Singapore) antibody diluted at 1:1000 in PBS and Anti-MRTF-A (Goat, sc-21558, Santa Gruz Biotechnology) antibody diluted at 1:100 in PBS for overnight at 4 °C. Then cells were washed three times with 0.05% Triton X-100 in PBS and incubated with Alexa Fluor 488 Donkey Anti-Rabbit IgG Antibody (A-21206, Molecular Probes), Alexa Fluor 647 Chicken Anti-Goat IgG Antibody (A-21469, Molecular Probes) and rhodamine–phalloidin (200 units in DMSO, R415, Molecular Probes) diluted at 1:1000 in PBS for 30 min. DNA was stained with 4', 6-diamidino-2-phenylindole (DAPI; 0.2 mg/ml in PBS, D9542, Sigma-Chemical Co.) at room temperature for 5 min. Images were acquired on a Delta Vision System (Applied Precision) centered on an Olympus IX70 microscope and equipped with a CoolSNAP HQ^2 CCD camera (Photometrics, Tucson, AZ).
